# Do not neglect the indigenous peoples when reporting health and nutrition issues of the socio-economically disadvantaged populations in Malaysia

**DOI:** 10.1186/s12889-019-8055-8

**Published:** 2019-12-16

**Authors:** Geok Lin Khor, Zalilah Mohd Shariff

**Affiliations:** Department of Nutrition and Dietetics, Faculty of Medicine and Health Sciences, Universiti Putra Malaysia, 43400 Serdang, Selangor Malaysia

**Keywords:** Indigenous peoples, *Orang Asli*, Health, Nutrition, Food security, Low-income, Malaysia

## Abstract

The purpose of this correspondence is to express our disappointment with the coverage of the BMC Public Health supplement: Vol 19 (4) titled “*Health and Nutritional Issues Among Low Income Population in Malaysia*”, which neglected to include the fundamental health and nutrition issues that are adversely affecting the lives and livelihood of the indigenous peoples. The Supplement comprised 21 papers. Two of these papers included indigenous peoples as study subjects. These two papers addressed peripheral, albeit important health issues, namely visual impairment and quality of life, and not the persistent and rising health concerns impacting this population. We will provide evidence from research and reports to justify our critique that the Supplement missed the opportunity to spotlight on the serious extent of the health and nutritional deprivations of the indigenous peoples of Malaysia. As researchers of the indigenous peoples, we ought to lend our voice to the “silenced minority” by highlighting their plight in the media including scientific journals.

## Main text

According to the lead Supplement Editor, the BMC Public Health supplement titled “*Health and Nutritional Issues Among Low Income Population in Malaysia*”, “presents work by the Consortium of Low Income Population Research (CB40R), highlighting a comprehensive aspect of health, i.e., physical health, mental health, health behaviour and health financing; and also nutrition involving all stages of lifespan of the socioeconomic deprived group in Malaysia” [1]. The stated aim of this Consortium is “to develop a system/framework of minimum/standard variables to be collected in research involving B40 in future”.

In Malaysia the country population is categorised into three different income tiers: Top 20% (T20), Middle 40% (M40), and Bottom 40% (B40), based on median household income [2]. The values of the median household income, which may increase or decrease year-to-year, depending on the country’s gross domestic product (GDP), serve as one of the indicators of the country’s economic growth.

It is also the stated position of the lead Supplement Editor that “All the papers in this special issue have successfully highlighted the health and nutritional issues (i.e., noncommunicable disease (NCD), inflammatory bowel disease (IBD), knowledge towards sexually transmitted disease (STD), low birth weight, Motoric Cognitive Risk (MCR) syndrome, urinary incontinence), mental health, oral health and inequalities among the low-income group in Malaysia, including the rural population and also the urban poor” [1].

Whilst we do not dispute the importance of these health issues affecting the rural and urban poor, we are concerned that the BMC Public Health readers might misconceive that the population groups covered by the Supplement are the only low-income or socio-economically disadvantaged populations in Malaysia. We wish to correct any misrepresentation that might arise, owing to the failure of the Supplement to address the fundamental health and nutrition issues that are adversely affecting the lives of the indigenous peoples in Malaysia. We are disappointed that the Supplement did not highlight the health and nutrition issues of the indigenous peoples, who are reportedly among the most socio-economically disadvantaged population groups in Malaysia, in terms of access to basic health care, food and nutrition security [3, 4].

The Supplement comprised 21 papers, out of which 2 included indigenous peoples as study subjects. These two papers addressed peripheral, albeit important health issues, namely visual impairment and quality of life^1^, and not the persistent and rising health concerns impacting this population. Both of us, the authors, have been conducting research and community work on public health and nutrition aspects of the indigenous peoples in Malaysia for decades [5–7]. We will provide evidence from research and reports to support our critique that the Supplement missed the opportunity to spotlight on the serious extent of the health and nutritional deprivations of the indigenous peoples.

## Who are the indigenous peoples of Malaysia? A brief overview

In 2015, it was estimated that collectively, the indigenous peoples of Malaysia represented about 13.8% of the country’s total population of 31.7 million [8]. The indigenous peoples do not belong to a homogenous population group. On the contrary, they consist of several different ethno-linguistic groups living in Peninsular Malaysia and in the states of Sarawak and Sabah in east Malaysia.

1. In Peninsular Malaysia, the indigenous peoples are referred to as *Orang Asli* (“original peoples” in Malay), a collective term for its three main groups, namely the “*Negritos*”, “*Senoi*” and the “*Proto-Malays*”, each of which has various sub-ethnic groups [9].
2. In Sarawak, the *Dayaks* is the collective term used for several native groups. The *Dayaks* account for 40% of the population of Sarawak. The “*Iban*” and the “*Bidayuh*” are the largest groups within the *Dayak* community.
3. In Sabah, there are some 39 different indigenous groups constituting almost 60% of the state population. The “*Kadazan-Dusun*”, “*Bajau*” and “*Murut*” are the largest indigenous groups in Sabah.

## Focus on the *Orang Asli*

Given the numerous indigenous population groups in the country, we wish to focus on the *Orang Asli* in Peninsular Malaysia, based on the vast body of research evidence that showed the persistence of environmental and health problems affecting the *Orang Asli* community. Moreover, the Director-General of the Department of *Orang Asli* Affairs had recently reported that 99% of *Orang Asli* “subsists on a level that is below the government’s classification of poor income [10]. As it is the stated aim of the consortium (CB40R) to collect research information on the B40, we believe that according attention on the *Orang Asli* will provide useful materials for the future work of the Consortium.

The *Orang Asli* have lived amid decades-long violations of land rights, including encroaching commercial interests like logging, mining and plantations into their traditional territories, lack of infrastructure, access roads, clean water supply, electricity as well as education, health and medical care. Any of these factors, acting singly or in combination, might have contributed to the recent occurrence of the sudden deaths of several *Orang Asli* (*Batek* sub-tribe), which captured headlines, such as this report in The Guardian [11]:

**Malaysia’s last indigenous nomadic tribe threatened by deadly mystery illness**

*“Over the past month, 14 people from the tribe’s village in north-eastern Peninsular Malaysia died, and more than 50 have been taken to hospital. Some are still in intensive care and more are being admitted by the day. Forty-seven others have been treated for respiratory problems. The source of the illness in this small village of 300 indigenous people, known as the Orang Asli– a Malay phrase meaning “original people” – remains unknown. Local authorities have cordoned off the area as they investigate, testing for everything from infectious disease to pollution or poisoning from nearby mines, and have begun exhuming the buried bodies for post mortems”.*

## No shortage of narratives and scientific discourses on the *Orang Asli*

There is substantial literature on the *Orang Asli* with writings going back 200–300 years to the early encounters of explorers, naturalists, missionaries and colonial administrators. Racial undertones and explicit disparaging names were patent in some of these initial narratives, such as the reference to the “wild tribes of the interior of the Malay Peninsula” by Wallace [12] and Borie [13], and the description of the “Pagan races of the Malay Peninsula” by Skeat and Blagden [14]. Nonetheless, these early reports, among many others, provided a wealth of historical, ethnographic, linguistic and cultural excerpts about the *Orang Asli* [15–17]. These works have been comprehensively reviewed by several writers, including Benjamin [18], Carey [19], Lye [20, 21], Nicholas and Baer [22] and Rusaslina [23].

By the mid-twentieth century, academic dissertations by scholars from Malaysia and overseas had expanded insights and knowledge on the *Orang Asli* beyond the traditional anthropological realms to a more heterogenous collection of works [5, 24–29]. “This literature ranges from studies of prehistory and human evolution to health, biomedicine and linguistics to the classic anthropological concerns for kinship, mythology, social organisation and environmental relations to, more recently, political analyses of development, assimilation, poverty and land rights” [21].

By the turn of the millennium, concomitant with the establishment of several institutions of higher learning in the country, and with increased public funding available for research, there has been an upsurge of research studies, including those on the *Orang Asli* from health, nutrition and food security perspectives.

## Studies on *Orang Asli* health, food and nutrition security

Despite the government poverty reduction strategies over the past decades to improve the livelihood of the *Orang Asli* [30], this indigenous population remains socio-economically marginalised. Persistence of a high prevalence of undernutrition, and on-going reports of mortality of preventable infectious disease like measles and malaria^2^ is a clear manifestation of serious shortfalls in the social and public health policies and programmes for the *Orang Asli*.

Childhood undernutrition is a persistent health problem in the *Orang Asli* population that has been documented over the decades at levels that are much higher than those reported in other rural or poor communities in Malaysia. Studies reported that child undernutrition remains relatively high (43–86%) with stunting being more prevalent than underweight [31–38]. Anaemia indicative of iron deficiency and helminthic infestation is also an age-old nutritional problem affecting the *Orang Asli* [5]. In general, short maternal stature, low birth weight, prematurity, low dietary diversity, parasitic infections, inadequate sanitation and hygiene are among the major prenatal and postnatal determinants of undernutrition among the *Orang Asli* children.

While underweight and stunting are common in *Orang Asli* children, overweight and obesity are becoming increasingly prevalent among the adults. Studies across the sub-tribes showed that approximately 10–50% *Orang Asli* adults were overweight and/or obese, with women more likely to be overweight or obese than men [39–43]. There is also accumulating evidence of metabolic risks (i.e. impaired glucose intolerance, hypertension, central obesity, hyperlipidaemia) in the *Orang Asli* adults [41–46]. The rising prevalence of metabolic risks could lead to increasing occurrence of diabetes mellitus, hypertension and cardiovascular diseases in the indigenous community.

The poor health and nutritional status of the *Orang Asli* children and adults could be attributed largely to food and nutrition insecurity affecting a majority of this population, particularly women and children [6, 40, 47–49]. The changing food systems of the *Orang Asli,* which could be due to the challenges of maintaining traditional food systems as well as increasing availability, accessibility and acceptance of westernized food, is partly responsible for the food and nutrition insecurity in this population. The consumption of cheaper energy-dense and nutrient-poor foods due to limited financial resources is likely to predispose children to poor growth and development, as well as providing excess calories for adults leading to obesity and its attendant chronic metabolic disease outcomes.

## Conclusion

The research evidence that we have shared point to a worsening of the health, nutrition and food insecurity conditions of the *Orang Asli*, particularly to the detriment of the women and young children in the community. Hence, it is imperative for researchers to give voice to the “silenced minority”^3^ by spotlighting their plight in the media including scientific journals. We therefore reiterate that the editors of the Supplement [1] missed an apt opportunity to speak out for the indigenous peoples of Malaysia.

## Data Availability

All the references cited in the manuscript are included in the Reference list.

## Abbreviations

B40: Bottom 40% based on median household income
CB40R: Consortium of Low Income Population Research
GDP: Gross domestic product
M40: Middle 40%
T20: Top 20%

## Footnotes

1 “*Status of visual impairment among indigenous (Orang Asli) school children in Malaysia*”, and “*Health related quality of life (HRQOL) among low socioeconomic population in Malaysia”,* respectively [1].

2 As of July 9, since the measles outbreak was reported in June, some 174 cases with symptoms and four deaths had been reported. Kelantan recorded 144 cases and three deaths, Terengganu (22 cases and one death) while Pahang had eight cases (https://www.thestar.com.my/news/nation/2019/07/09/health-ministry-confirms-fourth-orang-asli-death-due-to-measles)

3 “*The Aboriginal Peoples Act of 1954 is arguably the most important piece of legislation* *governing the lives of the Orang Asli in Malaysia today. This law governs all aspects of* *the Orang Asli’s lives, including land rights, education and who can be defined as Orang* *Asli. This law effectively sets up the Orang Asli as wards of the state and thus limits their* *rights as full citizens”* [18].
